# The Distribution of Cool Spots as Microrefugia in a Mountainous Area

**DOI:** 10.1371/journal.pone.0135732

**Published:** 2015-08-18

**Authors:** Ayuma Shimokawabe, Yuichi Yamaura, Takumi Akasaka, Tomonori Sato, Yuichiro Shida, Satoshi Yamanaka, Futoshi Nakamura

**Affiliations:** 1 Division of Environmental Resources, Graduate School of Agriculture, Hokkaido University, Sapporo, Hokkaido, Japan; 2 Faculty of Environmental Earth Science, Hokkaido University, Sapporo, Hokkaido, Japan; 3 Department of Forest Vegetation, Forestry and Forest Products Research Institute, Tsukuba, Ibaraki, Japan; 4 Laboratory of Wildlife Ecology, Obihiro University of Agriculture and Veterinary Medicine, Obihiro, Hokkaido, Japan; University of Colorado, UNITED STATES

## Abstract

It has recently been proposed that microrefugia played an important role in species survival during past climate change events. However, the current distributions of microrefugia remain largely unknown. Wind-hole sites are areas affected by preferential flows of cool air generated in interstitial spaces created by rock fragments or colluvia. Alpine plant species occurring in lowland wind-hole sites isolated from alpine zones may be relicts of the last glacial period. Hokkaido, northern Japan, is known to contain many wind-hole sites in which alpine plant species can occur. Here we surveyed 55 wind-hole sites in the Kitami region, eastern Hokkaido, and observed two alpine plant species (lingonberry, *Vaccinium vitis-idaea*, and Labrador tea, *Rhododendron groenlandicum* ssp. *diversipilosum* var. *diversipilosum*) in 14 wind-hole sites. Statistical modeling showed that wind-hole sites are likely to occur in areas with high maximum slope angles and volcanic rock cover, and concave surfaces. Our predictions of wind-hole site distributions suggest that such topographic conditions are common in our study area, and that many undiscovered wind-hole sites exist. Ignoring microhabitats may greatly underestimate species distributions in topographically complex regions, and dispersed cool spots may also function as stepping stones and temporal habitats for cold-adapted species. Because these localized unique habitats usually occur in economically unproductive sites, identifying and protecting potential microrefugia (cool spots) would be a robust and cost-effective mitigation of climate change impacts.

## Introduction

How the present and ongoing climate change event affects biodiversity is one of the most important topics in ecology. It is predicted that climate change may lead to range shifts and extinctions of many species, e.g., [1, 2, 3], but the accuracy of such claims is actively debated [4, 5]. One limitation is the use of coarse resolution data (e.g., around 100 km^2^) because of constraints on the availability of species distribution data, surface climates, and computer capacity [6, 7]. Recent studies have suggested that different conclusions are obtained upon fine-scale analyses. For example, locally suitable habitats may be overlooked in coarse analyses, which discount variations in topography and temperature [8–10]. Fine-resolution models of species distributions would greatly improve predictive accuracy in topographically complex regions [6, 11], and may enable us to identify overlooked ‘microrefugia’ [6, 12].

[13] defined a microrefugium as ‘a small area with local favorable environmental features, in which small populations can survive outside their main distribution area (the macrorefugium), protected from the unfavorable regional environmental conditions’. Studies have recently proposed that, along with macrorefugia, microrefugia played an important role in the survival of species during past climate change events [13–15]. It is suggested that species persisted in the dispersed microrefugia during the glacial periods, and expanded their distributions from the microrefugia in the warm periods [16]. However, microrefugia are no more than a concept in palaeoecology [13], and the distributions of microrefugia have not been empirically demonstrated.

In the current interglacial period, microrefugia for cold-adapted species are likely to be ‘cool spots’, which have lower temperatures than the surrounding environment [17]. Candidates for cool spots are local topographic depressions, heavily incised valley bottoms, and areas surrounding cool spring waters [16, 17]. In this study, we focused on “wind holes”, in which preferential flows of cool air are generated in interstitial spaces of rock fragments or colluvia, and therefore act as cool spots [18, 19]. There are many arguments on the mechanism of wind-hole formation. The strongest one is air convection generated by the difference in temperature between outer air and wind holes inside talus [20, 21]. In winter, air in wind holes is relatively warmer than outer air, which generates cold air penetration from wind holes at foot-slopes and warm air emission at upper slopes (anabatic airflow in wind holes). Conversely, in summer, air in wind hole is cooler than outer air, and therefore katabatic flow of cool air dominates in the holes [19, 21]. Localized permafrost in block slopes may partly contribute to the formation of wind holes [22]. Hereafter, areas affected by wind holes as referred to as ‘wind-hole sites’ [18, 20]. Unique vegetation is often established in wind-hole sites, where cool temperatures are locally maintained from spring to autumn [23]. In Hokkaido, northern Japan, alpine plant species such as lingonberry, *Vaccinium vitis-idaea* (Ericaceae), and Labroador tea, *Rhododendron groenlandicum* ssp. *diversipilosum* var. *diversipilosum* (Ericaceae), are found at wind-hole sites in lowland forested landscapes [20]; these may be relicts of the last glacial period [24]. Northern spider species also occur at wind-hole sites below the tree line in Europe [25]. Thus, lowland wind-hole sites harboring cold-adapted species may be microrefugia. However, the large-scale distributions of wind-hole sites are unknown.

Although there have been a few attempts to predict large-scale distributions of microrefugia [26], identifying such areas would enable us to discover overlooked microhabitats, and would contribute to our understanding of large-scale species distributions. Consideration of microrefugia may also change the predictions of climate change impacts and provide us with a chance to mitigate the impacts [27]. For example, fine-resolution models including microrefugia would prevent overestimates of extinction risk [16]. The importance of small localized unique habitats including wind-hole sites has been acknowledged in conservation biology (e.g., cliffs, caves, and serpentines), and called as keystone ecosystems, specialized habitats and geo-sites [28–30]. It is suggested that most landscapes incorporate such unique habitats, and conflicts between the protection of these habitats and commercial activities can be negligible [29]. [21] described the nation-wide distribution of wind-holes in Japan. Therefore, identification of microrefugia and subsequent their protection would lead to a cost-effective mitigation of climate change impacts in many landscapes (sensu [31]). Here, we surveyed wind-hole sites in lowland forested landscapes, and constructed statistical models describing the distributions of these sites. We also recorded the occurrences of alpine plant species at individual wind-hole sites, and we discuss the roles of wind-hole sites as microhabitats and microrefugia.

## Materials and Methods

### Study area

**Fig 1:**
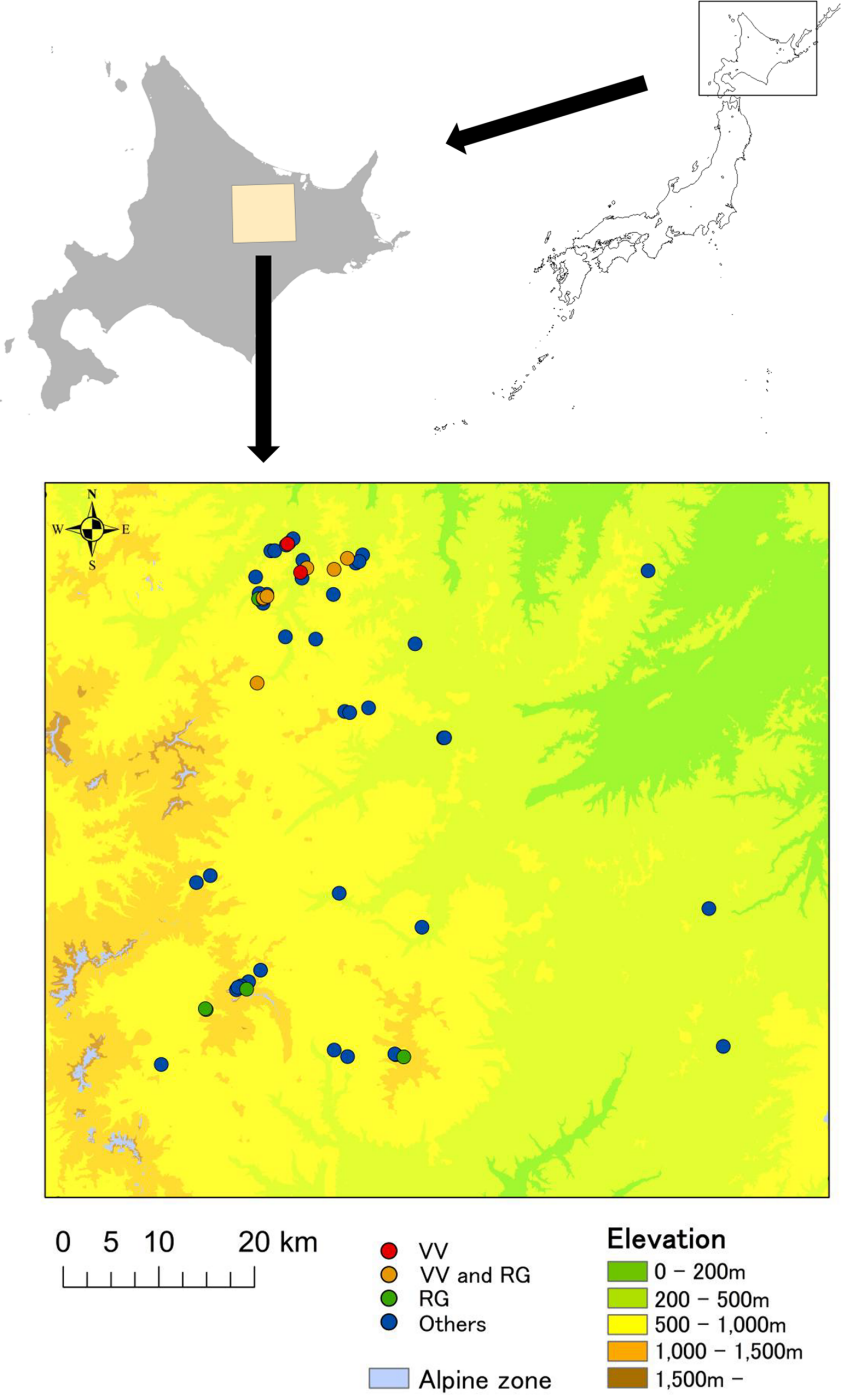
Study area and locations of wind-hole sites. We successfully located 55 wind-hole sites dispersed throughout the study area. Wind-hole sites with lingonberry (VV), Labrador tea (RG), both species, and without these species (others), are shown using different symbols. This figure is not identical to the original data, and is for representative purposes only.

Hokkaido is known to contain many wind-hole sites; we found 16 studies describing wind-hole sites in Hokkaido (e.g., [20, 21, 32]; listed in S1 Document), and they suggest that many wind-hole sites exist in the Kitami region, eastern Hokkaido, where we conducted field surveys (43°20’N-44°N, 143°E-144°E, Fig 1). In the western part of the study area, there are high mountains (1,500–2,000 m.a.s.l.), and alpine vegetation prevails above 1,500 m. The northeastern area is occupied by the basin of Kitami-city. Lower mountains and hills cover the other parts of the area.

### Field survey

The literature gives only the approximate locations of wind-hole sites. For example, only district names or coarse-resolution maps were available. We thus searched for both described and undescribed wind-hole sites in the study area between July to September 2012 and June to July 2013 (22 days in total). When searching for new wind-hole sites, we drove along roads in a car while a researcher in the passenger seat searched the landscape. We also frequently searched on foot. When we found accumulated debris containing vegetation different from that of the surrounding environment, we explored whether cold air flowed from debris gaps. Specifically, we placed burning incense at gap surfaces and observed the direction of the smoke. We measured the temperature 20 cm below the ground (soil temperature), at the surface, and at 120 cm above the ground (air temperature) using thermometers (Thermo Recorder RT-12, ESPEC MIC Corp., Aichi, Japan). The depth of debris is suggested to be dozens of meters [19, 33]. When air flow was observed and soil and/or ground surface temperatures were distinctly lower than the air temperature (≤ 7°C), we determined that these areas were wind-hole sites. We recorded the central coordinates of wind-hole sites using a geographic positioning system (GPS Pathfinder Pro XR, Nikon-Trimble Co., Ltd., Tokyo, Japan). We also recorded the presence/absence of lingonberry and Labrador tea. These are typical alpine species occurring at lowland wind-hole sites in Japan. All surveyed sites were under the jurisdiction of local forestry offices (western Abashiri, central Abashiri, eastern Tokachi, western Tokachi Forest Offices, and Okhotsk General Subprefectural Bureau), which provided the permission for our field survey.

### Modeling the occurrence probability of wind-hole sites

We examined the effects of environmental covariates on the probability of wind-hole site occurrence, using MAXLIKE [34]. This method treats occurrence probability as a function of environmental covariates using presence-only data; the module compares covariates between sites of presence and the entire region of interest. Unlike common statistical methods that evaluate presence-only data, MAXLIKE attempts to estimate an occurrence probability rather than a ‘relative’ occurrence probability, assuming a random sample. Because the implementation of MAXLIKE needed long time, we selected candidate covariates via ordinary logistic regression, and fitted MAXLIKE with selected covariates using R ver. 2.15.2 [35] and the R package ‘maxlike’ ver. 0.1–5 [36].

First, we divided the study area into 10-m resolution raster data using a 10-m digital elevation model (DEM: Geospatial Information Authority of Japan) and ArcGIS ver. 10 (ESRI Inc., Redlands, CA, USA). This resolution approximated the mean size of wind-hole sites (see results). We treated cells containing the centers of wind-hole sites as presence cells. Avoiding presence cells, we generated one million random pseudo-absence cells to use in logistic regression. Pseudo-absence cells can include unobserved presence cells, meaning that the analysis using pseudo-absence cells measures the deviation of presence cells from the background cells [37].

### Topographic and geological covariates and selection procedure

Candidate covariates were selected based on the topographic and geological formation processes of wind holes. Wind holes frequently occur at the bottoms of talus slopes where fragmented rocks accumulate and interstitial spaces are formed [21, 32]. Wind holes also occur on volcanic rocks such as rhyolite, andesite and welded tuff because these rocks have an inherent propensity to produce cobble sized debris, which form interstitial spaces [21, 32]. We used mean slope angle, mean slope angle squared, maximum slope angle, and mean curvature (positive, negative, and zero values indicate convex, concave, and flat curvature, respectively) as topographic variables. We used the mean slope angle squared because wind holes often feature medium slope angles. We also used “volcanic rock cover” consisting of rhyolite, andesite, and welded tuff because talus slopes with rock fragments and associated wind holes are typically found in these geological regions (see below).

We generated 10-m raster data of slope angles and curvatures from the DEM, this is termed the 0-m buffer-size data. We next generated circular buffers of different radii (25, 50, 100, 200, 300, 400, and 500 m) from individual cells, and calculated mean and maximum angles, and the mean curvature within buffers. We masked urban, farmland, and water areas from these calculations using a 1:50,000 vegetation map produced by the Biodiversity Center of Japan, Ministry of the Environment, Government of Japan. Random pseudo-absence data were generated from the unmasked area. We then fitted logistic regressions with each topographic variable of each buffer size as a single covariate, and used variables at the buffer sizes that yielded the smallest AICs upon final analysis. We always used the mean slope angle squared together with the mean slope angle. The mean slope and the square thereof that yielded the smallest AIC were used as covariates.

For geological variables, we first classified surface geology into volcanic rocks, and others, using a 1:200,000 surface geology map published by the Ministry of Land, Infrastructure, Transport and Tourism of Japan, which was rasterized at 10-m resolution. We again did not consider urban, farmland, or water areas. We generated circular buffers with 200-m radii from individual cells, considering the measurement error (± 136 m) of the map. We next created a categorical variable indicating whether volcanic rock had larger proportions or not (1/0), and a continuous variable indicating the proportion of volcanic rocks. We used the single variable with the lowest AIC in final analysis.

### Fitting MAXLIKE

To avoid multi-collinearity, correlation coefficients were calculated among selected covariates. The covariate with the lower AIC was selected when the correlation coefficient was > 0.7. Logistic models were constructed for all possible combinations of covariates, and the model with the lowest AIC was treated as the best model. We fitted MAXLIKE using the covariates in the best logistic model. To enhance convergence, we standardized covariates before implementing MAXLIKE. We calculated the explained deviance as an explanatory power of the fitted model (pseudo-R^2^): 1 –(D_f_ / D_null_) where D_f_ and D_null_ are deviances of fitted and null models, respectively. We obtained the deviance of the respective model by multiplying the log-likelihood by –2. We also obtained the explained deviance of the logistic regression model with random pseudo-absence data and the same explanatory variables. We further fitted the MAXLIKE model to the data only composed of wind-hole sites with alpine plant species.

## Results

### Field survey

**Fig 2:**
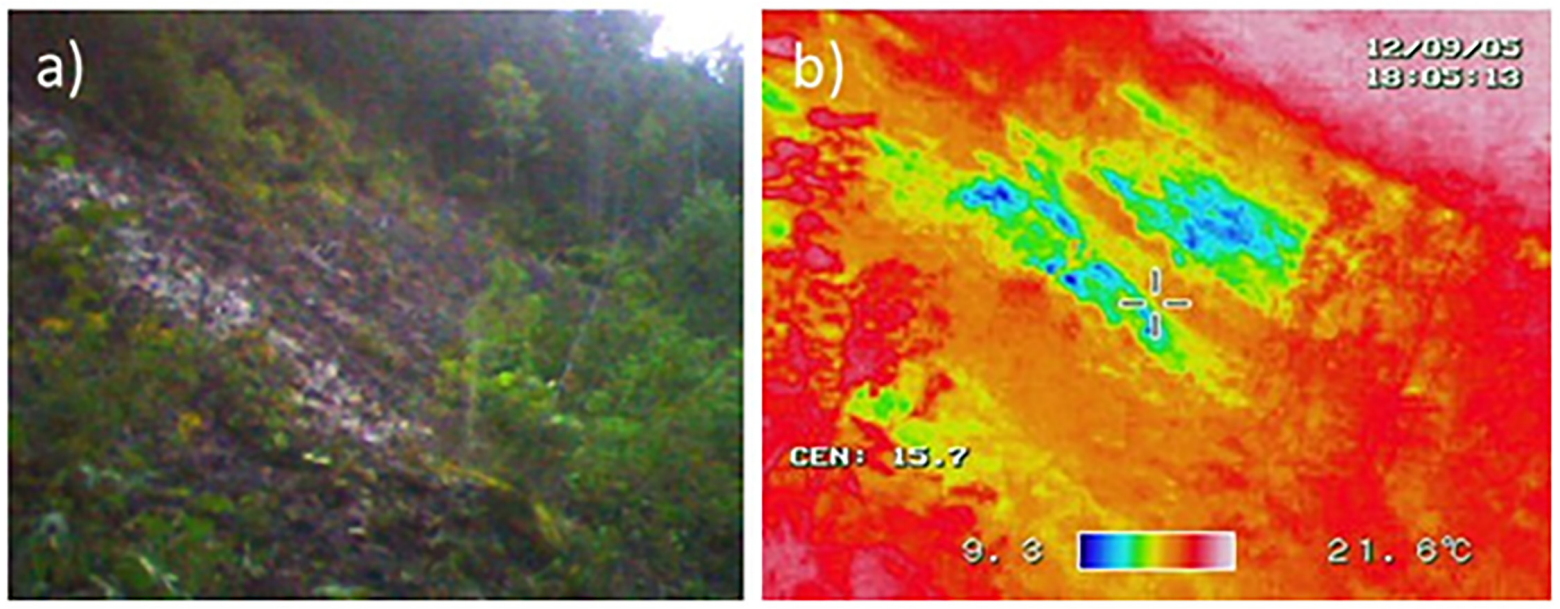
(a) A wind-hole site and (b) its surface temperature measured using a thermal camera. Areas with accumulated debris had lower temperatures.

We found 27 described and 28 new wind-hole sites (Figs 1 and 2). Although we did not measure their areas, small wind-hole sites were ~ 10 m^2^ in area, whereas large sites were > 2,000 m^2^ in area (mean area, ~100 m^2^). The sites were dispersed throughout the study area, and almost all were at the bottoms of slopes in the low elevations (230–1,181 m.a.s.l., mean 556 m). Lingonberry or Labrador tea occurred at 9 and 12 sites, respectively, and at least one plant occurred in 14 of the 55 sites. The 14 wind-hole sites with such alpine plants were also located at low elevations, far away from the alpine zone (elevation: 290–1,181 m.a.s.l., mean 600 m; distance: 0.4–19.5 km, mean 10.7 km; Fig 2).

### Statistical analysis

For mean slope angle, logistic models with a 100-m buffer containing both the mean value and the square thereof had the lowest AIC. Buffers of 100 m and 50 m were best when maximum slopes and curvature, respectively, were analyzed (Table 1). For surface geology, the continuous variable had a lower AIC. Because the square of the mean angle and the maximum angle were highly correlated (Table 2), we used only the maximum angle; this had the lower AIC value. The following three covariates were entered into the final model: maximum slope angle (100 m), curvature (50 m), and surface geology (continuous). We fitted logistic models with these covariates, and the model with all three covariates had the lowest AIC (Table 3).

**Table 1:** AIC values of logistic models for individual covariates.

Buffer size (m)	0	25	50	100
Mean slope angle	1191.7	1191.2	1187.9	1184.7
Mean slope angle squared	1190.0	1181.8	1159.6	**1154.9**
Maximum slope angle	1191.7	1175.1	1145.3	**1143.7**
Curvature	1182.3	1164.5	**1120.7**	1123.5
Buffer size (m)	200	300	400	500
Mean slope angle	1183.6	1182.0	1180.8	1180.8
Mean slope angle squared	1161.1	1159.4	1161.1	1163.0
Maximum slope angle	1159.4	1151.4	1150.2	1153.1
Curvature	1128.5	1139.4	1166.9	1165.1
Surface geology (categorical)	1182.9			
Surface geology (continuous)	**1179.0**			
Null model: 1190.9				

Values with the 0-m buffer were based on calculations generated without buffers. Bold text denotes the lowest AIC for each covariate. For mean slope and its square, the single model with the lowest AIC was selected.

**Table 2:** Correlation matrix of selected covariates.

	MeanSA^2^	MaxSA	Curv.	Geol.
Mean slope angle^2^ (100 m)	1			
Maximum slope angle (100 m)	0.827	1		
Curvature (50 m)	-0.002	-0.005	1	
Surface geology (continuous)	-0.030	-0.033	0.017	1

Covariate names in columns are abbreviated.

**Table 3:** Results of model selection using logistic regressions.

	Parameter estimates					
Model #	Intercept	MaxSA	Curv.	Geol.	AIC	ΔAIC
8	-13.610	0.07148	-1.232	1.339	1085.6	0.00
6	-12.600	0.06702	-1.227		1103.0	17.32
4	-11.170		-1.861	1.268	1105.6	19.92
2	-10.350		-1.817		1120.7	35.05
7	-14.380	0.10010		1.303	1127.5	41.84
5	-13.330	0.09443			1143.7	58.05
3	-10.540			1.150	1179.0	93.39
1	-9.808				1190.9	105.26

See Table 2 for abbreviated parameter names.

**Fig 3:**
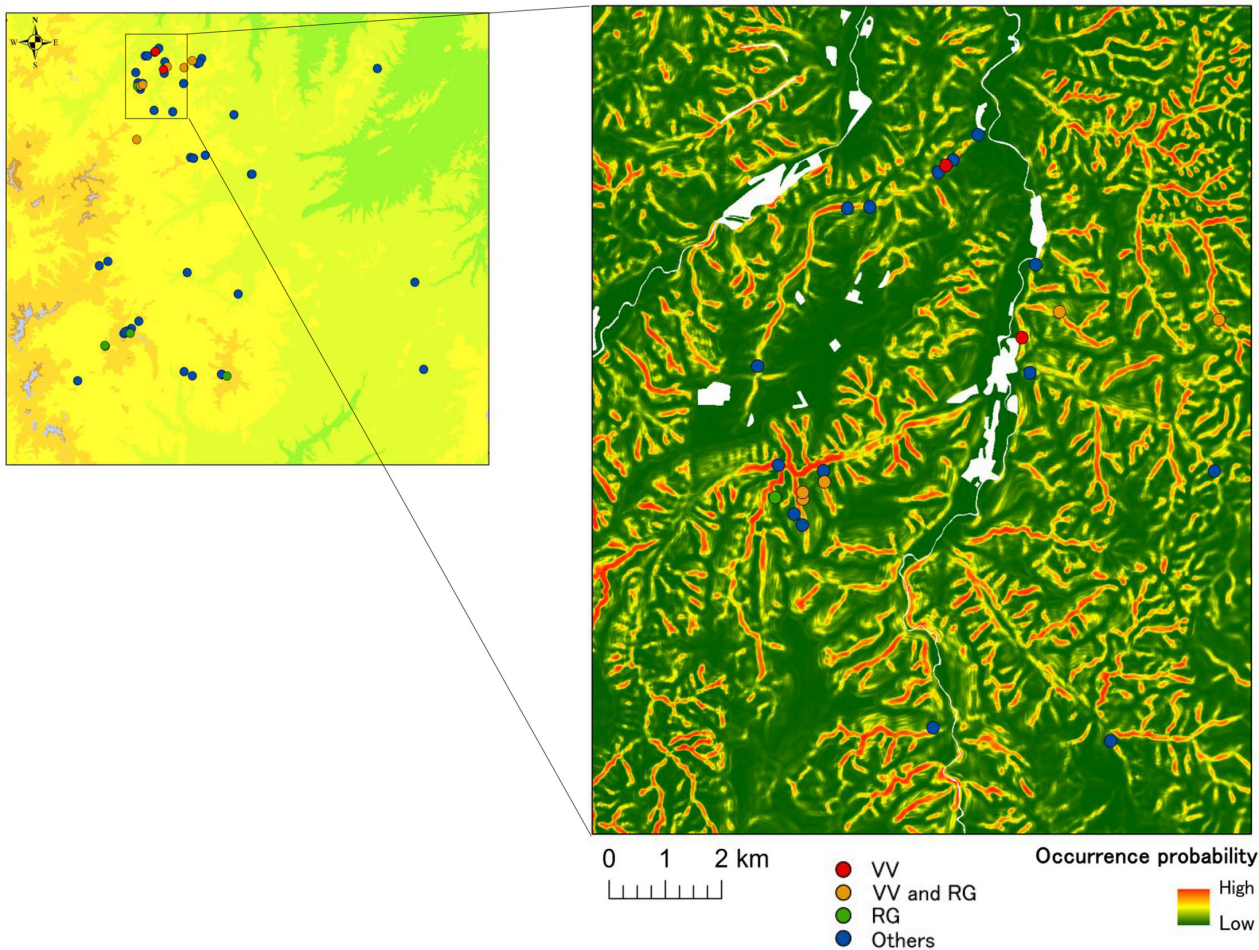
Predicted occurrence probabilities of wind-hole sites in part of the study area. See Fig 1 for an explanation of symbols.

Model fitting with MAXLIKE showed that the effects of maximum slope angle, curvature, and surface geology were significant (Table 4A). Model estimates suggest that the probability of occurrence increases with increasing maximum slope angle, proportion of volcanic rocks, and concave surfaces. However, the explained deviance was low (0.07), and presence cells included low values of predicted occurrence probability (0.975, 0.75, 0.5, 0.25, and 0.025 percentiles of occurrence probabilities for presence cells were 0.99, 0.72, 0.44, 0.14, and 0.03, respectively; mean predicted value was 0.46). The comparable model of the logistic regression model had the similar value of the explained deviance (0.09). We used this MAXLIKE model to predict wind-hole site occurrences in the study area (Fig 3). As parameter estimates suggest, cells at the bottoms of steep slopes had higher occurrence probabilities. We also fitted the MAXLIKE to the data of 14 sites with alpine plants, and the results were similar with the former 55 site model except that the effect of curvature was lessened (Table 4B). But parameter uncertainties were larger. The explained deviance was also similar with that of the former model (0.06).

**Table 4:** Estimates of MAXLIKE model coefficients.

Parameter	Estimate	SE	*z*	*P*(>|*z*|)
(a) 55 site model				
Intercept	-3.687	0.487	-7.57	< 0.001
Maximum slope angle (100 m)	1.393	0.396	3.52	< 0.001
Curvature (50 m)	-1.517	0.423	-3.59	< 0.001
Surface geology (continuous)	0.963	0.279	3.46	< 0.001
(b) 14 site model				
Intercept	-4.605	1.921	-2.40	0.0165
Maximum slope angle (100 m)	1.238	0.559	2.22	0.0267
Curvature (50 m)	-0.572	0.337	-1.70	0.0894
Surface geology (continuous)	1.320	0.527	2.50	0.0123

Note that covariates were standardized before analysis.

## Discussion

To our knowledge, this is the first study to construct empirical statistical models of large-scale wind-hole site distributions. We obtained the precise locations of wind-hole sites using a field survey, and tested the effects of topographic and geological covariates on the probability of wind-hole site occurrence. As supported by their formation processes [21], we found that wind-hole sites are likely to occur in areas with high maximum slope angles and volcanic rock cover, and concave surfaces. Absolute values of standardized model coefficients were slightly larger for topographic than for geological covariates (Table 4), suggesting that the effects of topographic covariates were larger. However, it is possible that geological effects were underestimated because of the coarseness of geological source data. Explanatory power of the model was small, and the model predicted low occurrence probabilities for wind-hole sites. Therefore, there would be additional factors relevant to the wind-hole formations such as larger-scale region-wide topography (see below).

Statistical models enabled us to predict the large-scale distributions of wind-hole sites. As model parameters suggest, sites at the bottoms of steep slopes had higher probabilities of containing wind holes. [33] found the presence of cold air pool in the valley bottom (sensu [38]) in Kitami region in winter. This cold air pool may further contribute to lower the air temperature inside the wind-hole sites. The predictive map indicates that such topographic conditions are common in our study area, suggesting that there are many undiscovered wind-hole sites. Indeed, we discovered 28 undescribed new wind-hole sites during our field survey. Nevertheless, it is difficult to estimate ‘absolute’ occurrence probabilities using presence-only data [39–41]; we can only conclude that wind-hole site occurrences depend on topographic and geological covariates. Collection of absence data, fine-grained geological maps, and process-based parameters of wind-hole formations, would improve our understanding and predictions of large-scale wind-hole site distributions.

Two alpine plant species (lingonberry and Labrador tea) occurred at low-elevation wind-hole sites that were remote from alpine zones (Fig 2). Wind-hole sites likely provide alpine species with suitable microhabitats and contribute to the distributions of these species. It is noted that the surroundings of the wind-hole sites were well forested, and we observed these two alpine plant species exclusively in wind-hole sites at low elevation areas. Our results suggest that ignoring local habitats can underestimate species distributions in topographically complex regions. However, fewer than half of the wind-hole sites (14 of 55) contained these alpine plants (Fig 2), and the model only for 14 sites with alpine plant species was slightly different with that of all wind-hole sites (Table 4), suggesting that curvature may not be so important to the distribution of alpine species. However, parameter uncertainties were large in 14-site model, and this suggestion was not conclusive. Another potentially candidate factor explaining the restrictive distribution of alpine plant species was their distributions during the last glacial period. It would be difficult for alpine species to colonize sites isolated from past alpine zones. If we can show that alpine species have persisted in wind-hole sites since the last glacial period, we can term such sites microrefugia. Therefore, additional approaches such as genetic studies are needed to test this hypothesis.

If cold temperatures are maintained in wind-hole sites during the present climate change event, other cold-adapted species may be able to persist in these microhabitats. [33] suggested the possible longevity (more than two thousand years) of wind-hole sites in Hokkaido. The distributions of many species are predicted to change because of a changing climate [1–3]. Possible microrefugia, including wind-hole sites, may save species from extinction via short-distance migrations [16]. In this respect, we suggest the potential conservation values of cool spots currently without alpine plant species. Cool spots may be wide-spread [17, 21], as shown in this study, and function as stepping-stones and temporal habitats (holdouts) for cold-adapted species in general, especially those of taxa with high dispersal ability (sensu [15]). Because these localized unique habitats usually occur in economically unproductive sites [29], identifying and protecting potential microrefugia (cool spots) would be a robust and cost-effective mitigation of climate change impacts (sensu [31]), and may be more so than other mitigation schemes (e.g., assistance of long-distance migration).

## Supporting Information

S1 Document Literature describing existing wind-hole sites. (DOCX)

S1 Fig Wind-hole sites in central Hokkaido, northern Japan. Wind-hole sites (a) with accumulated rocks and (b) covered by lingonberry.
(DOCX)
