# Supplementary material for: The Distribution of Cool Spots as Microrefugia in a Mountainous Area
Source: PLoS One. 2015 Aug 18;10(8):e0135732. doi: 10.1371/journal.pone.0135732 (PMC4540282; doi:10.1371/journal.pone.0135732)
Supplement: S1 Document — (DOCX) [file pone.0135732.s001.docx]

**S1. Literatures describing the existing wind-hole sites.**

1. Adachi, H. Permafrost in the Abashiri region, Hokkaido. Komazawa Geography. 1974; 10:61-72.
2. Ishikawa, Y, Nobetsu, T. Research on the state of altherbosa and wind-swept site community along the coast of Shiretoko. Report of Shiretoko world natural heritage site ecosystem monitoring in 2006. 2007; 105-116. Shiretoko Nature Foundation.
3. Samejima, J, Shiozaki, M, Kishida, A, Sanada, M, Samajima, K. Forest vegetation in Akan. The nature of Akan national park Volume 1. 1993; 468-526. Maeda ippoen foundation.
4. Sato, K., Kudo, G, Uemura, S. Cool-spots site vegetation in Izariiri-Heide, northern Japan. Japanese Journal of Ecology. 1993; 43: 91-98.
5. Sato, T, Uchida, A, Hayashi, M, Hayashi, H, Obana, Y, Hayashi, H. Species assembly of pteridophytes at Engaru, northeastern Hokkaido: focusing a disjunctive distribution of Cystopteris sudetica in Hokkaido and Nagano. Bulletin of the Shiretoko Museum. 2011; 32: 9-18.
6. Shiboi, T. Periglacial geomorphic phenomena observed in Kitami District. Memoirs of The Kitami Institute of Technology. 1973; 4(2): 303-320.
7. Shiboi, T. On the periglacial geomorphic phenomena observed in the Johmon Ridge area: mechanism of cold anomalies. Memoirs of The Kitami Institute of Technology. 1974; 5(2): 213-242.
8. Shiboi, T. On the subsurface cold air circulation observed at Onneyu-tsutsujiyama, Rubeshibe-cho, Hokkaido. Journal of Geography. 1974; 83: 89-102. doi: 10.5026/jgeography.83.2_89.
9. Shiboi, T. Periglacial geomorphic phenomena observed in the Yubetsu River terrain, including the ice-shove ridge along the beach of Okhotsk. Memoirs of The Kitami Institute of Technology. 1975; 6(2): 139-159.
10. Shiboi, T. Supplementally report on the periglacial phenomena observed in Kitami District. Memoirs of The Kitami Institute of Technology. 1975; 7 (1): 163-194.
11. Shiboi, T. Cold anomalies and underground ice in Kitami District. Bulletin of Hokkai-gakuen Kitami University. 1980; 3: 141-152.
12. Shimizu, C. An information on the cool air blow holes (wind-holes) including the ice caves in Japan: references to the surrounding landforms and the existence of sporadic permafrost. Komazawa Geography. 2004; 40: 121-148.
13. Shimizu, C. A list of wind holes in Japan. Geography. 2009; 54(7): 76-81.
14. Shimizu, C, Yamakawa, N. Frozen ground at the mountain slopes around the Higashi Taisetsu area, central Hokkaido, in the summer of 2000. Bulletin of The Higashi Taisetsu Museum of Natural History. 2001; 23: 21-31.
15. Sone, T. Degradation of extra-zonal permafrost near Kanoko dam, Oketo town, Hokkaido, northern Japan. Quarterly Journal of Geography. 1996; 48: 293-302. doi: 10.5190/tga.48.293.
16. Tokachi Research Group. 14C-Age of the sporadic permafrost of the Tokachi-mitsumata, Hokkaido: 14C-Age of the Quaternary Deposits in Japan (90). Earth Science. 1973; 27(6): 258-260.
