# Supplementary material for: The Distribution of Cool Spots as Microrefugia in a Mountainous Area
Source: PLoS One. 2015 Aug 18;10(8):e0135732. doi: 10.1371/journal.pone.0135732 (PMC4540282; doi:10.1371/journal.pone.0135732)
Supplement: S1 Fig — Wind-hole sites (a) with accumulated rocks and (b) covered by lingonberry. (DOCX) [file pone.0135732.s002.docx]

**S2. Wind-hole sites in central Hokkaido, northern Japan.**


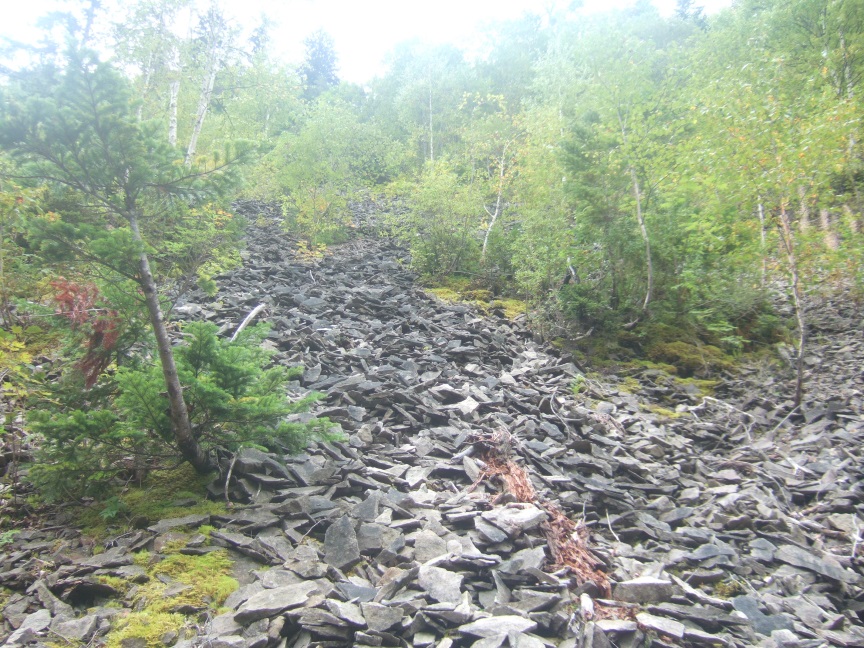


**(a)**


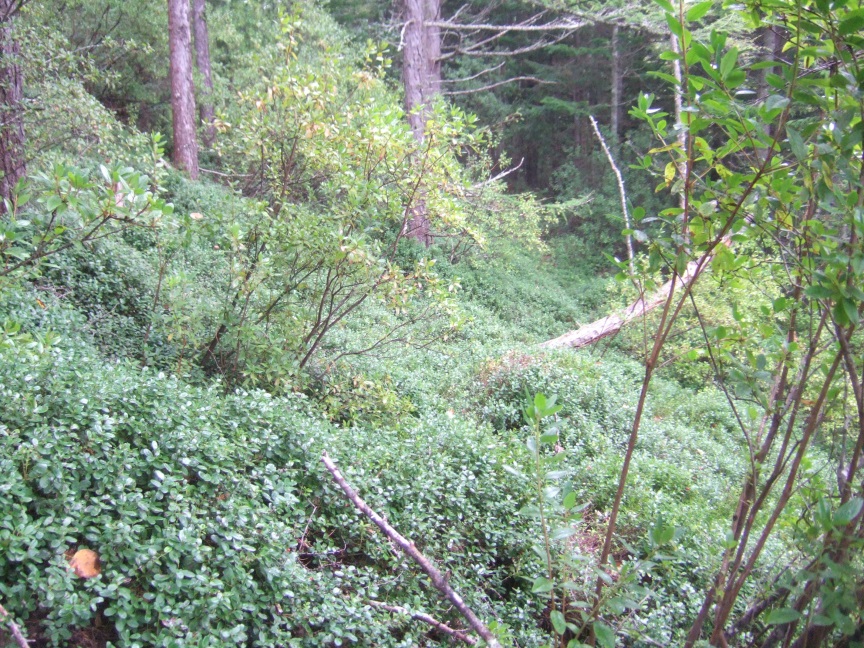


**(b)**

Wind-hole sites (a) with accumulated rocks and (b) covered by lingonberry.
